# Biomarkers of RV Dysfunction in HFrEF Identified by Direct Tissue Proteomics: Extracellular Proteins Fibromodulin and Fibulin-5

**DOI:** 10.1161/CIRCHEARTFAILURE.124.011984

**Published:** 2025-01-17

**Authors:** Matěj Běhounek, Denisa Lipcseyová, Ondřej Vít, Petr Žáček, Pavel Talacko, Zuzana Husková, Soňa Kikerlová, Tereza Tykvartová, Peter Wohlfahrt, Vojtěch Melenovský, Jan Beneš, Jiří Petrák

**Affiliations:** 1 First Faculty of Medicine (M.B., D.L., O.V., J.P.), Biotechnology and Biomedicine Center of the Academy of Sciences and Charles University (BIOCEV), Charles University, Prague, Czech Republic.; 2 Proteomic Core Facility, Faculty of Science (P.Z., P.T.), Biotechnology and Biomedicine Center of the Academy of Sciences and Charles University (BIOCEV), Charles University, Prague, Czech Republic.; 3 Center of Experimental Medicine (Z.H., S.K.), Institute for Clinical and Experimental Medicine (IKEM), Prague, Czech Republic.; 4 Department of Cardiology (T.T., P.W., V.M., J.B.), Institute for Clinical and Experimental Medicine (IKEM), Prague, Czech Republic.; 5 Division of Cardiovascular Medicine, University of Utah School of Medicine, Salt Lake City (J.B.).

**Keywords:** biomarkers, extracellular matrix, fibromodulin, fibrosis, heart failure

## Abstract

**BACKGROUND:**

Right ventricular dysfunction (RVD) is common in patients with heart failure with reduced ejection fraction, and it is associated with poor prognosis. However, no biomarker reflecting RVD is available for routine clinical use.

**METHODS:**

Proteomic analysis of myocardium from the left ventricle and right ventricle (RV) of patients with heart failure with reduced ejection fraction with (n=10) and without RVD (n=10) who underwent heart transplantation was performed. Concentrations of 2 ECM (extracellular matrix) proteins with the highest myocardial upregulation in RVD, FMOD (fibromodulin) and FBLN5 (fibulin-5), were assayed in the blood and tested in a separate cohort of patients with heart failure with reduced ejection fraction (n=232) to test for the association of the 2 proteins with RV function and long-term outcomes.

**RESULTS:**

Multivariable linear regression revealed that plasma concentrations of both FMOD and FBLN5 were significantly associated with RV function regardless of the RV function assessment method. No association of FMOD or FBLN5 with left ventricular dysfunction, cardiac index, body mass index, diabetes status, or kidney function was found. Plasma levels of FMOD and FBLN5 were significantly associated with patient outcomes (*P*=0.005; *P*=0.004). Area under the curve analysis showed that the addition of FBLN5 or FMOD to RV function assessment had a significantly higher area under the curve after 4 years of follow-up (0.653 and 0.631, respectively) compared with RV function alone (0.570; *P*<0.05 for both). Similarly, the combination of MAGGIC (Meta-Analysis Global Group in Chronic Heart Failure) score, FBLN5, and FMOD had a significantly larger area under the curve (0.669) than the combination of MAGGIC score+RVD grade (0.572; *P*=0.02). The Kaplan-Meier analysis demonstrated that patients with the elevation of both FMOD and FBLN5 (ie, FMOD >64 ng/mL and FMOD >27 ng/mL) had a worse prognosis than those with the elevation of either FBLN5 or FMOD (*P*=0.03) demonstrating the additive prognostic value of both proteins.

**CONCLUSIONS:**

Our study proposes that circulating levels of FMOD and FBLN5 may serve as new biomarkers of RVD in patients with heart failure with reduced ejection fraction.

**WHAT IS NEW?**

- Heart failure–associated changes in myocardial protein expression can be identified by proteomics.
- Differentially expressed myocardial proteins can be used as disease markers, pending their altered expression in myocardium translates into their changed concentration in blood.
- ECM (extracellular matrix) proteins FMOD (fibromodulin) and FBLN5 (fibulin-5) are significantly upregulated in the myocardium and in plasma in patients with heart failure with reduced ejection fraction with right ventricular dysfunction.

**WHAT ARE THE CLINICAL IMPLICATIONS?**

- Currently, there is no specific circulating biomarker available for routine clinical use to recognize specifically right ventricular dysfunction in the context of heart failure with reduced ejection fraction.
- FMOD and FBLN5 plasma levels are strongly associated with right ventricular dysfunction and bring improved information about prognosis, which makes them promising biomarkers of right ventricular dysfunction.

Right ventricular dysfunction (RVD) is a major complicating condition of heart failure (HF) with both preserved and reduced ejection fractions and is associated with poor prognosis.^1–3^ Although no specific RVD-targeted therapy is currently available, RVD is commonly present in advanced HF and may herald clinical worsening with the need for therapy intensification or timely indication of heart transplantation. Development of severe RVD may preclude the use of long-term left ventricular (LV)–assist devices and, thus, considerably impacts long-term survival.^4^

The right ventricle (RV) function is currently assessed mainly by imaging, and echocardiography is the most commonly used method. There is no specific circulating biomarker available in routine clinical use to recognize specifically RVD in the context of HF with reduced ejection fraction (HFrEF). Identification of such a biomarker could add to echocardiographic RV evaluation, better risk-stratify patients, and help with further decision-making in advanced HFrEF management.

RVD may result from or translate into specific proteome changes in both ventricles. The proteins with altered expression can be used as disease markers, pending their altered expression in myocardium translates into altered concentration in blood. Such proteins can be either passively released from the myocardium as a result of tissue leakage caused by cell death or injury or actively secreted from the cells to the interstitial fluid. To look for such potential markers of RVD in patients with advanced HF, we have used proteomic analysis of myocardium from both ventricles of patients with HFrEF with and without RVD. Two proteins of ECM (extracellular matrix) showing the most marked upregulation in myocardial tissue were further assessed in plasma; their plasmatic levels were tested for the relationship with RV function and outcome.

## Methods

The data that support the findings of this study are available from the corresponding author upon reasonable request. The mass spectrometry data have been made publicly available by deposition to the ProteomeXchange Consortium via the PRIDE partner repository with the dataset identifier PXD043768.

All methods are described in detail in the Supplemental Material.

### Patients

Subjects with symptomatic but hemodynamically stable HFrEF (LV ejection fraction [LVEF] <40%) that had a duration of at least 6 months were enrolled in the study.^1,5^ Those with potentially reversible LV dysfunction (planned valve surgery, revascularization, or tachycardia-induced cardiomyopathy) were excluded. All research was performed in accordance with relevant guidelines/regulations, the protocol was approved by the Ethics Committee of the Institute for Clinical and Experimental Medicine (IKEM), and all subjects signed an informed consent.

### Patients Cohort 1 (Proteomics Cohort)

A cohort of 122 consecutive patients who underwent heart transplantation without preceding use of an LV-assist device was divided according to the RV function measured by fractional area change determined by a single experienced echocardiographer before heart transplantation. A subgroup of 10 HFrEF male patients with severe RVD (first quartile of fractional area change), and 10 body size, cause, age, and comorbidity-matched HFrEF male patients with preserved RV function (fourth quartile of fractional area change) were used for proteomic analysis. In addition, 10 age and body size–matched subjects without a clinical history of HF and with normal LV and RV functions that were refused as heart donors due to hemodynamic instability or moderate coronary lesions were added to the analysis as a proxy for nonfailing controls. Patient data for the proteomics cohort are provided in Table S1.

### Patients Cohort 2 (Clinical Cohort)

A group of 232 patients with HFrEF that were referred to IKEM for evaluation of advanced therapies, and 65 non-HF controls were used for this analysis. Patients were prospectively enrolled between 2017 and 2020 and underwent complex in-hospital evaluation, including echocardiography, right heart catheterization, and blood sampling. Patients were prospectively followed for the occurrence of an adverse outcome that was defined as the combined end point of death, urgent heart transplantation, or ventricular assist device implantation. Because the time to nonurgent transplantation reflects donor availability rather than the recipient’s condition, patients who received a nonurgent heart transplant were censored as having no outcome event on the day of transplantation. The control group consisted of 65 subjects (36 subjects without evidence of coronary artery disease or HF evaluated for the presence of patent foramen ovale; 29 subjects were healthy hospital employees).

## Results

### Proteomic Analysis

Myocardial samples of LV and RV from 10 patients with HFrEF and severe RVD, 10 patients with HFrEF and preserved RV function (noRVD), and 10 control individuals were subjected to quantitative proteomic analysis. Because RVD may be also associated with molecular changes in the LV, and with respect to their different ontology, wall thickness, working pressures, physiology, and pathology, we analyzed separately both RVs and LVs. Despite the matching for age, body, size, HF cause, and comorbidities, patients with severe RVD had shorter duration of HF, lower LVEF, and higher NTproBNP (all suggesting more rapid HF progression) than those with preserved RV function. The description of patients and controls is given in Table S1.

**Figure 1.**
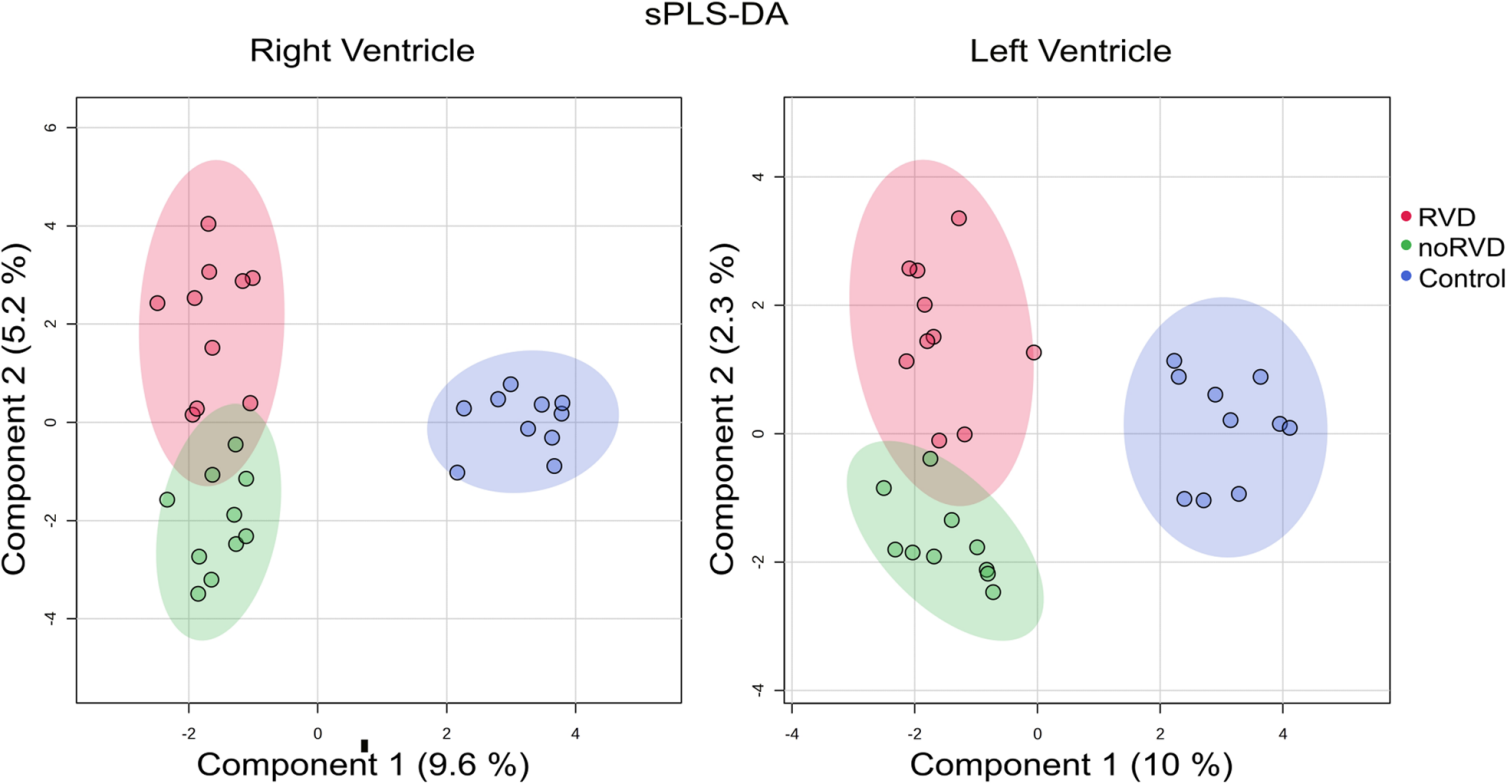
**Sparse least squares discrimination analysis (sPLS-DA) of the quantitative proteomic data.** Results of the separate analyses of the right and left ventricles are shown. RVD indicates right ventricular dysfunction.

**Figure 2.**
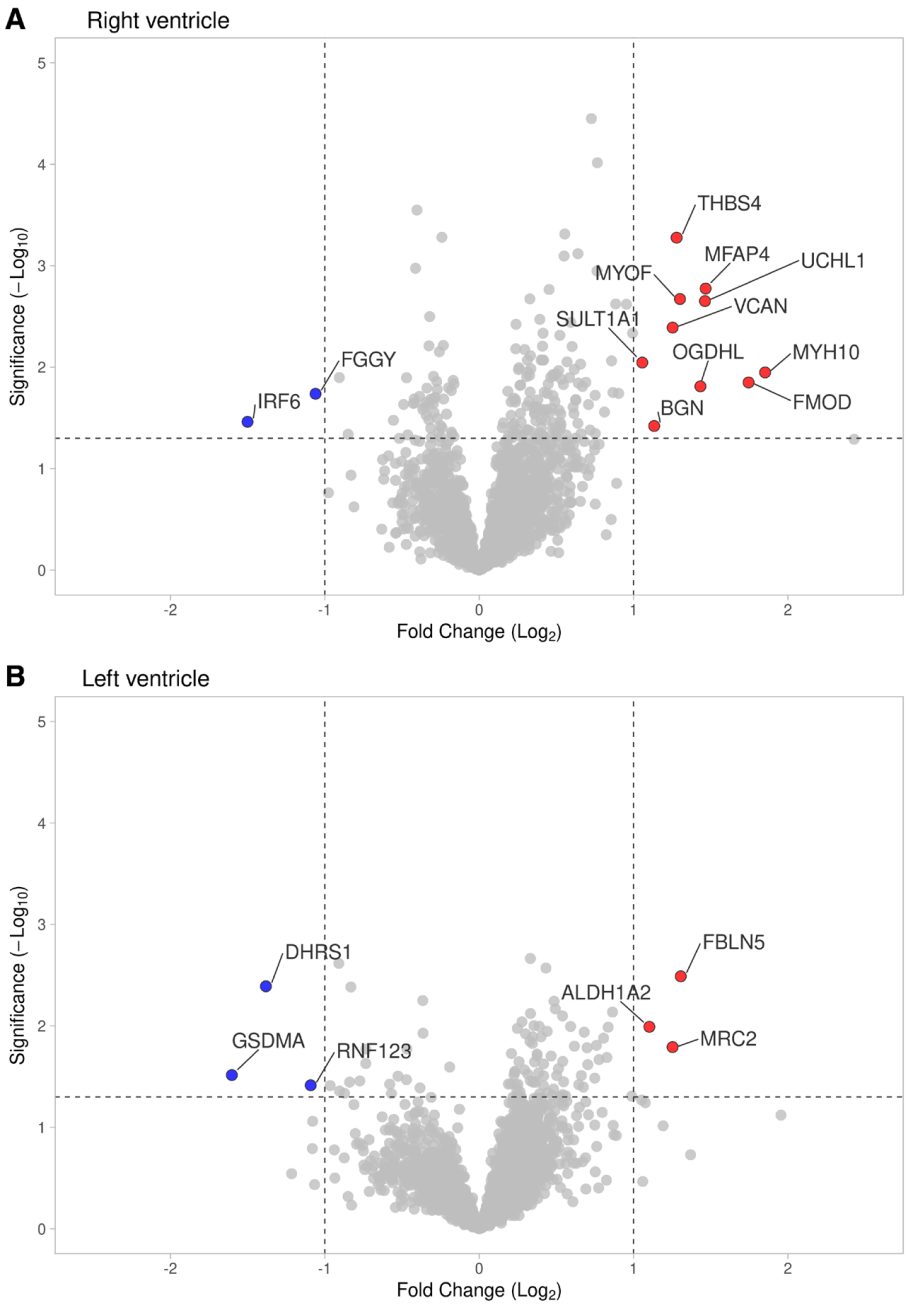
**Label-free quantification proteomic analyses of ventricular myocardium of patients with heart failure with reduced ejection fraction with right ventricular dysfunction (RVD) and without RVD (noRVD).** Proteins upregulated in RVD vs noRVD are shown in red and downregulated in blue. Dotted lines indicate the thresholds for a 2-fold change in expression (vertical) and statistical significance (*P*_adjusted_=0.05; horizontal). Gene names are shown instead of the full-length protein names. **A**, Protein expression changes in the right ventricle. **B**, Protein expression changes in the left ventricle.

The label-free quantification proteomic analyses of RV and LV resulted in the identification of 3477 and 3633 protein groups, respectively. Supervised discrimination analysis (sparse partial least squares discrimination analysis), of the proteomic data from RV and LV clearly separated nonfailing (control) hearts from the failing hearts and, albeit less completely, also samples from patients with RVD (red) and patients without RVD (green) in both ventricles (Figure 1). This incomplete separation of RVD and noRVD proteomic profiles reflects rather mild changes in the myocardial proteome of patients with HFrEF with RVD and without RVD. In fact, we identified only 12 and 6 differentially expressed proteins (>2-fold; *P*<0.05) in the RV and LV of patients with RVD, respectively, compared with patients without RVD (Figure 2). None of the 18 RVD-associated changes in protein expression was identified simultaneously in both ventricles. Table 1 lists the identified differentially expressed proteins. A list of all proteins identified in the proteomic analysis is available as a Supplemental Dataset. The mass spectrometry data have been made publicly available by deposition to the ProteomeXchange Consortium via the PRIDE partner repository with the dataset identifier PXD043768.

**Table 1.**
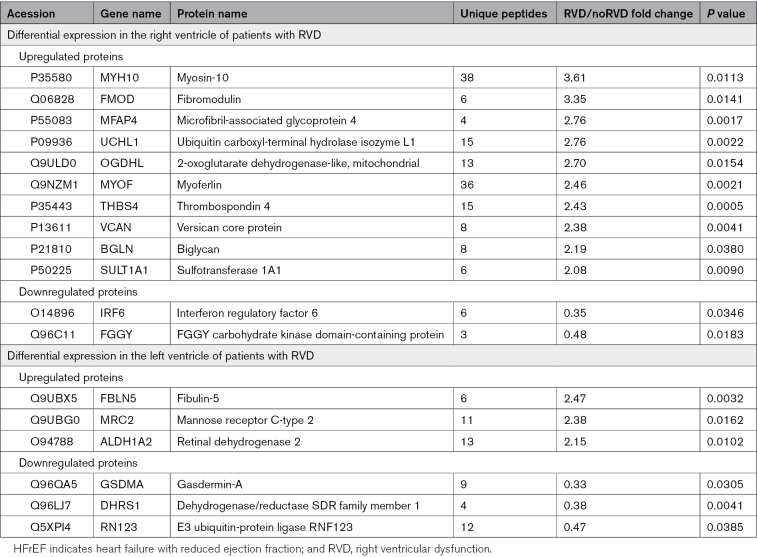
Proteins Differentially Expressed in Both Ventricles of Patients With HFrEF With RVD Compared With Patients With HFrEF Without RVD

### Bioinformatic Analysis-Pathway Enrichment

Because the proteome changes were limited and the numbers of the differentially expressed (>2-fold) proteins were low, gene ontology pathway analysis revealed significant enrichment only among the proteins upregulated in RV (Figure S1). “ECM organization” (*P*_adjusted_<0.001) was the process with the highest number of matched proteins.

In agreement with the gene ontology enrichment analysis, 5 of the 10 proteins that were markedly upregulated in the RV in patients with HFrEF with RVD (FMOD [fibromodulin], MFAP4 [microfibril-associated glycoprotein 4], VCAN [versican], BGLN [biglycan], and THBS4 [thrombospondin 4]) are ECM proteins annotated as “secreted,” “extracellular space,” and “ECM” according to the UniProt database. Similarly, the protein most markedly upregulated in the LV of patients with HFrEF with RVD, FBLN5 (fibulin-5), is also annotated as secreted/ECM protein. In addition, 3 other proteins upregulated in the hearts of patients with RVD, namely, MYH10 (myosin-10), UCHL1 (ubiquitin carboxyl-terminal hydrolase isoenzyme L1), and MRC2 (C-type mannose receptor 2), are known to participate in the process of ECM remodeling as well, despite their intracellular localization.^6–8^ The results of our proteomic analysis, thus, clearly pointed toward pronounced ECM remodeling as a hallmark of RVD in patients with HFrEF.

### Confirmation of the Proteomic Data in Myocardial Tissue

**Figure 3.**
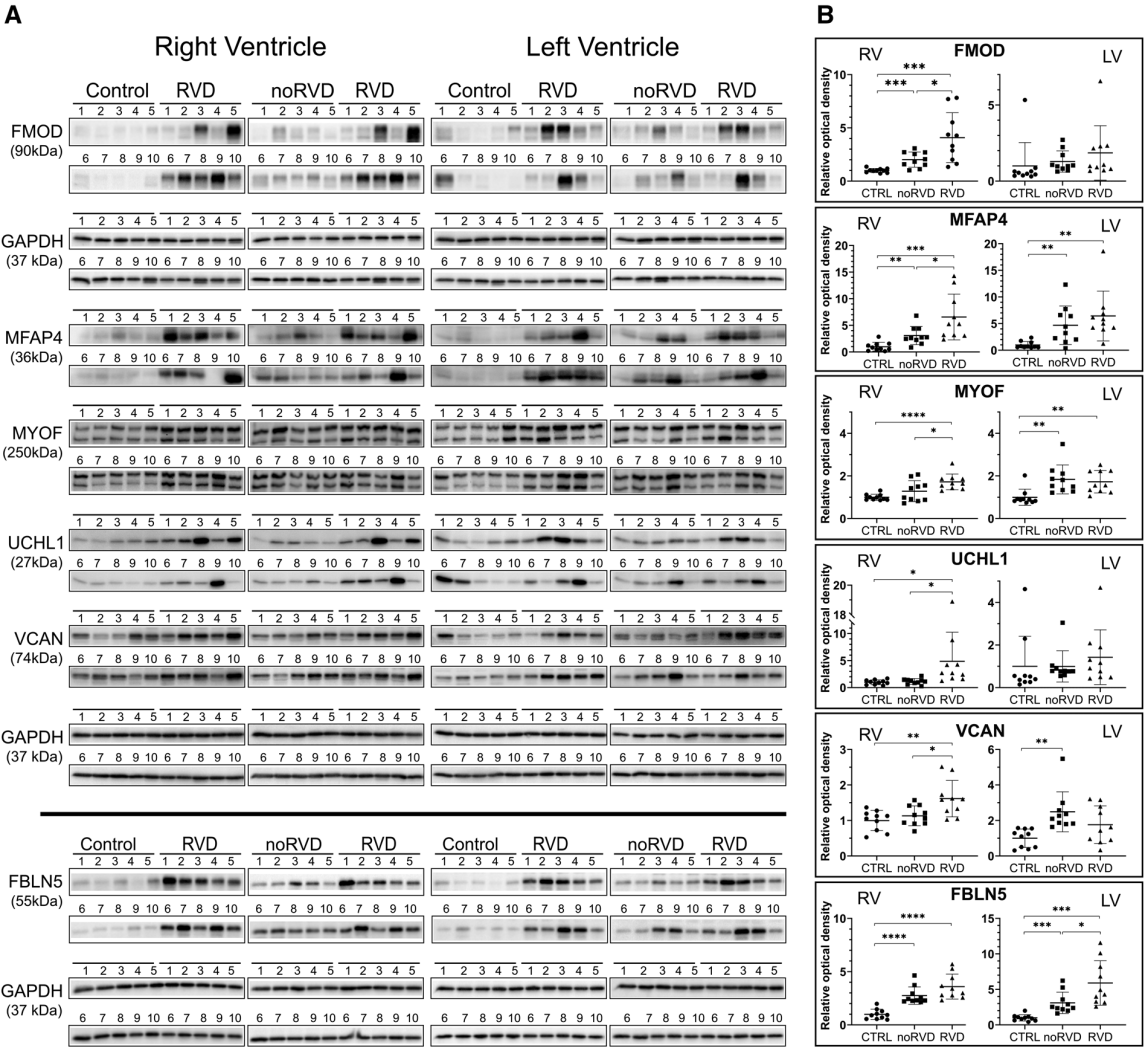
**Confirmation of differential expression by Western blotting. A**, Relative protein expression was determined in both ventricles of all the patients included in the proteomic analysis (right ventricular dysfunction [RVD] and noRVD). To obtain also information on the background expression, nonfailing donor heart samples were included (control [CTRL]). Right ventricular (RV) and left ventricle (LV) samples were analyzed separately; GAPDH was used as a loading control. **B**, Densitometry of the Western blots. The relative optical density of the bands normalized to the CTRLs is plotted. Statistical significance is indicated where reached. Gene names are shown instead of full protein names FMOD (fibromodulin), MFAP4 (microfibril-associated glycoprotein 4), VCAN (versican), MYOF (myoferlin), UCHL1 (ubiquitin carboxyl-terminal hydrolase isoenzyme L1), and FBLN5 (fibulin-5).

The differential myocardial expression of 6 proteins discovered using our proteomic approach was subsequently verified using specific antibodies. The same samples were used for both the proteomic and confirmatory analyses. The results of the confirmatory analysis were fully in agreement with the proteomic analysis, and the expression of FMOD, MFAP4, UCHL1, MYOF, and VCAN was confirmed to be significantly higher in the RV of HFrEF patients with with RVD, while the expression of FBLN5 was significantly increased in the LV (in HFrEF patients with RVD). All the proteins were also significantly upregulated relative to the respective ventricles of nonfailing controls (Figure 3).

### Secreted/ECM Myocardial Proteins in Blood Plasma

Five of 10 proteins upregulated in the RV of patients with RVD are secreted proteins, components of ECM, and we reasoned that secreted proteins could serve as circulating biomarkers. FMOD was the most markedly (3.3-fold according to the proteomic analysis) upregulated ECM protein in the RV of patients with HFrEF with RVD. FBLN5 was the most strongly upregulated protein (2.5-fold) in the LV of patients with HFrEF with RVD. Looking for RVD markers, we, therefore, asked whether the marked myocardial upregulation of these secreted/ECM proteins would be mirrored in the blood plasma of patients with HFrEF. Proteins from both ventricles were considered because both ventricles may be the source of (potentially distinct) information on RVD.

### FMOD and FBLN5 in the Blood Plasma

Using specific sandwich ELISA, we first determined plasma concentration of FMOD and FBLN5 in plasma samples of the 20 HFrEF individuals (collected at the time of the transplantation) represented in the proteomic analysis (the cohort 1) and healthy male volunteers of comparable age (n=10). Both FMOD and FBLN5 were detectable in the plasma of all analyzed groups (ie, controls, patients with HFrEF with preserved RV function, and patients with HFrEF with severe RVD).

Controls showed the lowest level of both FMOD (17.58±3.84 ng/mL) and FBLN5 (54.32±15.78 ng/mL), followed by patients with HFrEF with preserved RV function (22.19±8.18 ng/mL for FMOD and 71.63±23.61 ng/mL for FBLN5). Patients with HFrEF with RVD showed the highest concentrations for both proteins (27.14±8.86 from FMOD and 96.32±48.08 ng/mL for FBLN5; Figure S2). In agreement with the increased myocardial expression, the plasma concentration of both proteins in the RVD group was higher compared with controls (*P*=0.005 for FMOD; *P*=0.017 for FBLN5) and tended to be numerically higher compared with the noRVD group as well. Considering the limited size of the original cohort 1, we evaluated the plasma concentration of FMOD and FBLN5 in a large cohort of well-characterized patients with advanced HF (cohort 2).

### The Utility of FBLN5 and FMOD in Clinical Scenarios

We tested the utility of FMOD and FBLN5 plasma concentrations in a cohort of 232 patients with HFrEF and 65 controls. Patients (aged 56.75±10.32 years; 89.2% men) presented with advanced HF (86.2% New York Heart Association III/IV and mean LVEF 23.3%). Significant number of patients (n=142; 61.7%) had moderate or severe RVD (details in Table 2). FMOD and FBLN5 plasma levels progressively and significantly increased with worsening RV function (*P*_for trend_<0.0001 for both proteins). When we analyzed only patients with HFrEF, both proteins still showed a significant association with RVD although it was less significant for FBLN5 (*P_for trend_*<0.0001 for FMOD; *P_for trend_*=0.037 for FBLN5).

**Table 2.**
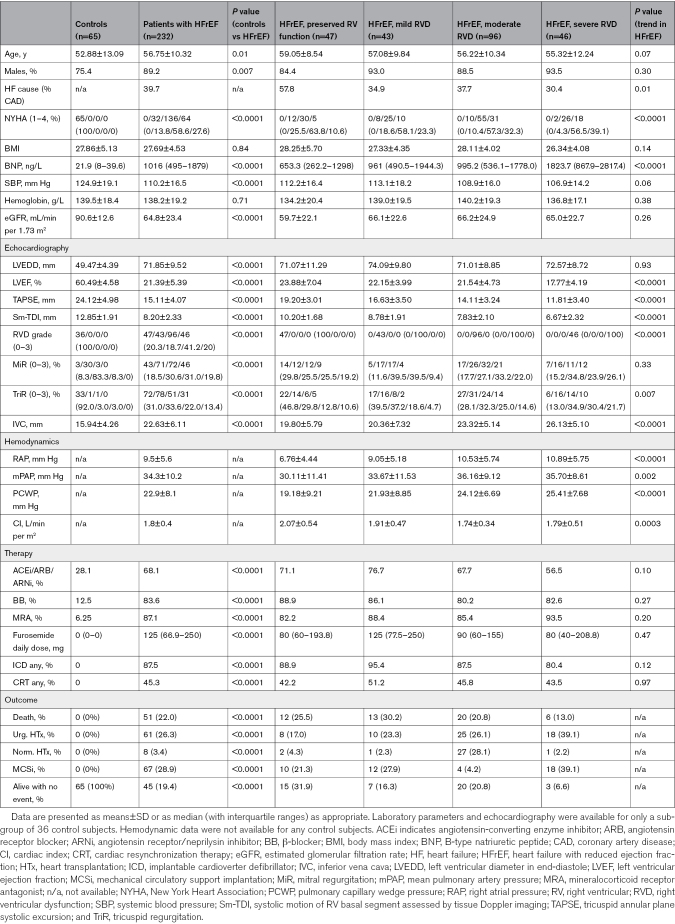
Clinical Cohort Patient Characteristics

### Regression Analysis

Despite the positive association of both FBLN5 and FMOD with RVD grade, we strived for a more robust analysis to answer the question of whether FBLN5 and FMOD are specifically associated with RVD or whether the observed association is due to possible confounders. We have performed multivariable regression analysis including most relevant confounders (LVEF, age, sex, body mass index, estimated glomerular filtration rate, the presence of diabetes, cardiac index, and pulmonary vascular resistance). RV function was assessed in 5 different ways (RVD grade, tricuspid annular plane systolic excursion, systolic motion of RV basal segment assessed by tissue Doppler imaging, pulmonary artery pulsatility index, and central venous pressure/pulmonary capillary wedge pressure ratio). Both FBLN5 and FMOD consistently showed significant association with RV function regardless of how RV function was assessed (Table 3). Besides the RV function, FBLN5 showed a consistent association with pulmonary vascular resistance and age. FMOD was strongly and significantly associated with RV function only.

**Table 3.**
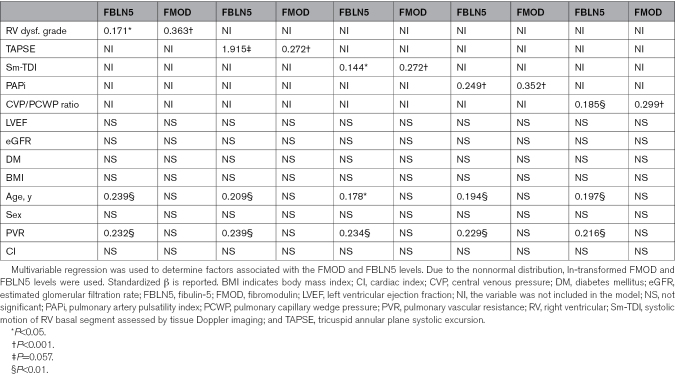
Regression Analysis

### FMOD and FBLN5 as Predictors of Adverse Outcome in HFrEF

During a follow-up of a median period of 4.4 (interquartile range 3.6–5.0) years, 179 patients (77.2%) in our cohort experienced an adverse outcome, defined as death/urgent heart transplantation/implantation of mechanical circulatory support. The Cox proportion hazard model revealed that both FMOD and FBLN5 were significantly associated with patient outcome, with a hazard ratio of 1.20 (95% CI, 1.05–1.37) per 1 SD of FMOD plasma concentration (*P*=0.005) and a hazard ratio of 1.30 (95% CI, 1.12–1.51) per 1 SD of FBLN5 plasma concentration (*P*=0.0004).

### Clinical Value of FMOD and FBLN5 Plasma Levels in Addition to RV Function Assessment

As both candidate markers FMOD and FBLN5 were associated with patient outcome and showed a significant association with RV function, we asked whether any of these proteins could improve the prognostic stratification of patients beyond RV function assessment by echocardiography. When adjusted for RVD grade, FMOD was no longer a significant predictor of outcome in Cox regression analysis (hazard ratio, 1.29 [95% CI, 0.98–1.30] for each SD of FMOD concentration; *P*=0.08). In contrast, FBLN5 remained significantly associated with outcome even when adjusted for RVD grade (hazard ratio, 1.30 [95% CI, 1.12–1.51] for each SD of FBLN5 concentration; *P*=0.0006).

Both proteins, however, showed a significantly higher area under the curve (AUC) when combined with RVD grade at a single time point of 4 years (176 of a total of 179 outcome events were reached at this time point; Table 4).

**Table 4.**
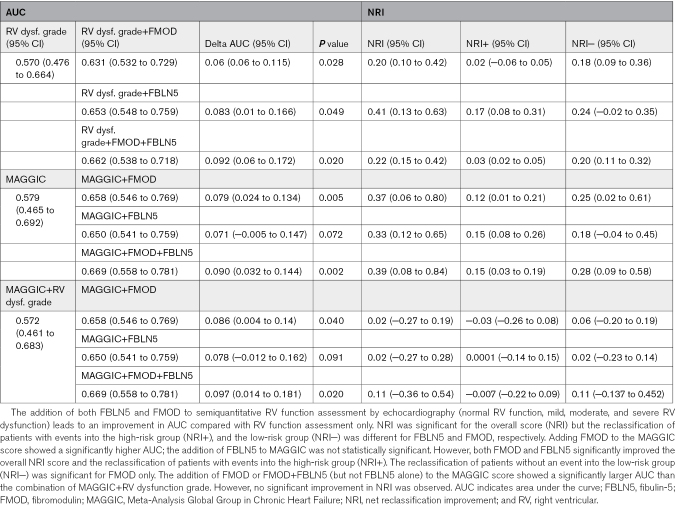
The Comparison of RV Function Assessment/MAGGIC Score With Addition of FMOD and FBLN5

We further used the net reclassification improvement (NRI) index that attempts to quantify how well a new model reclassifies subjects, either appropriately or inappropriately, compared with an old model.^9^ FBLN5 markedly improved the continuous NRI, with a significant improvement in event NRI (NRI+), but without a change in nonevent NRI (NRI−). FMOD markedly improved continuous NRI and nonevent NRI (NRI−) without a significant improvement of event NRI (NRI+; Table 4).

### Adding FMOD and FBLN5 to MAGGIC Score Improves Long-Term Prediction

In clinical practice, the prognosis of patients with HF is often evaluated by composite scoring systems that help to guide therapeutic decisions. MAGGIC score is one of those and encompasses various patient characteristics, degree of LV dysfunction (LVEF), HF pharmacotherapy, and comorbidities.^10^ We evaluated whether plasma concentrations of FMOD or FBLN5 add any additional prognostic information beyond the MAGGIC score (Table 4). Adding plasma FMOD to the MAGGIC score showed a significantly higher AUC after 4 years (*P*=0.005). In addition to that, FMOD correctly reclassified both patients with events into a more high-risk group (NRI+) and those without events into the low-risk group (NRI−). Adding plasma FBLN5 to the MAGGIC score numerically increased AUC; however, the change was not statistically significant (*P*=0.07).

Finally, we analyzed whether FBLN5 and FMOD added to the MAGGIC score can outperform MAGGIC together with RV function assessment. The addition of FMOD or FMOD+FBLN5 (but not FBLN5 alone) to the MAGGIC score resulted in a significantly larger AUC after 4 years than the combination of MAGGIC+RVD grade. Nevertheless, no significant improvement in NRI was observed (Table 4).

### A Combined Prognostic Role of FMOD and FBLN5

As both FMOD and FBLN5 plasma levels were found to be significantly associated with outcome, we analyzed the prognostic role of both proteins individually and when combined together.

**Figure 4.**
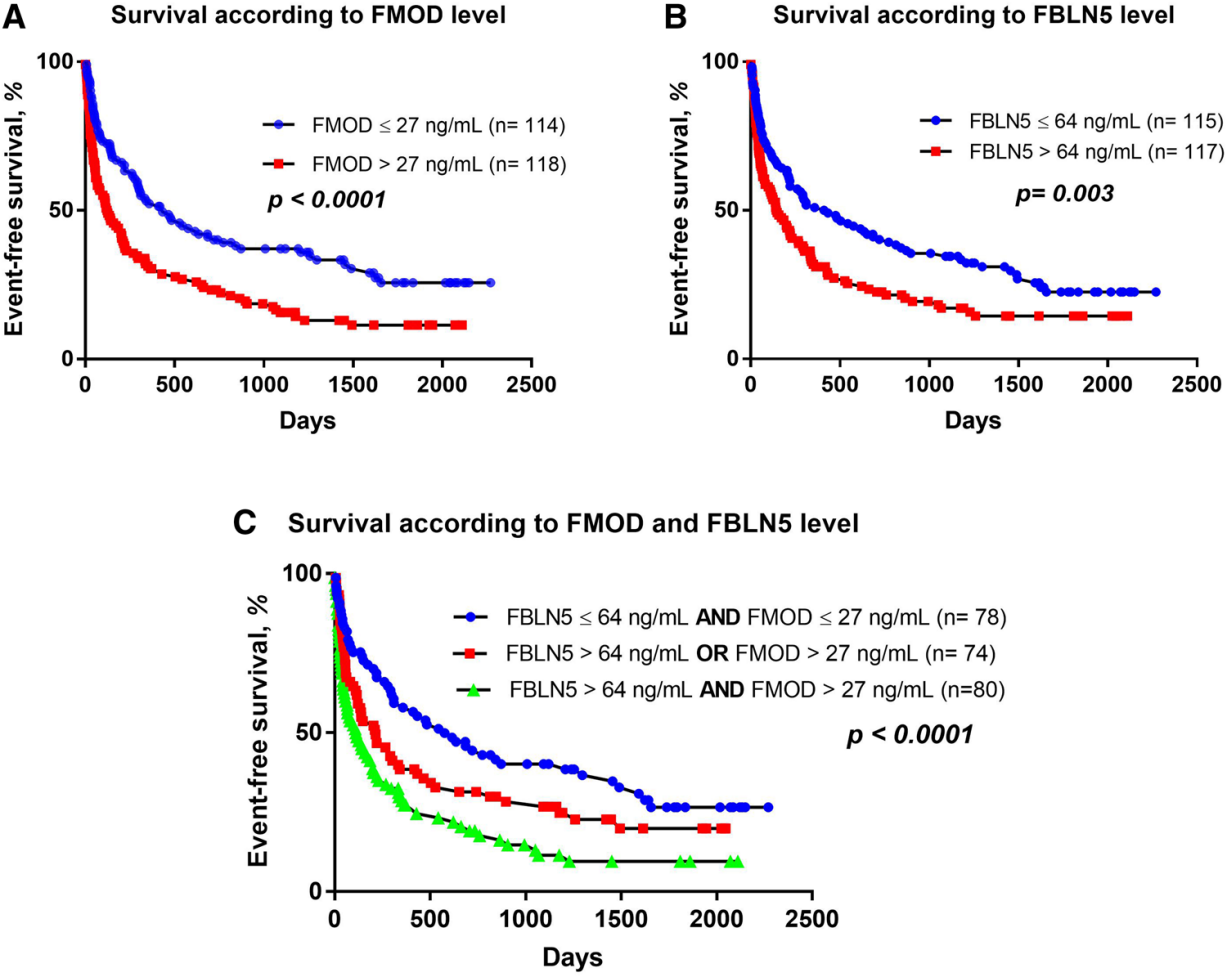
**Survival analysis according to the plasma levels of FMOD (fibromodulin) and FBLN5 (fibulin-5).** Patients were divided according to the mean plasma concentrations of FMOD and FBLN5. **A**, Kaplan-Meier analysis showing that patients with FMOD plasma concentration >27 ng/mL had worse outcomes than the rest of the cohort. **B**, Kaplan-Meier analysis showing that patients with FBLN5 concentration >64 ng/mL had worse outcomes than the rest of the cohort. **C**, Kaplan-Meier analysis showing the additive prognostic effect when both markers were elevated.

Patients were divided according to the mean plasma concentration of both proteins (ie, >27 ng/mL for FMOD and >64 ng/mL for FBLN5). Those with elevated concentrations of either FMOD or FBLN5 had worse event-free survival than the rest of the cohort (Figure 4A and 4B). When combined together, patients with an elevated concentration of either biomarker had significantly worse outcomes than those with low levels of both biomarkers. Similarly, patients with an elevation of both biomarkers had significantly worse outcomes than those with only 1 elevated biomarker (Figure 4C).

## Discussion

RVD is a commonly accompanying condition in patients with HFrEF and is associated with poor prognosis.^3,11^ Unfortunately, no specific biomarker of RVD in the HF context is available for routine clinical use. In our study, we have shown that highly sensitive and unbiased proteomic methods can assist with the identification of candidate marker proteins and that circulating concentrations of ECM proteins FMOD and FBLN5 may serve as indicators of RVD and predict the outcome of patients with HFrEF.

RVD is associated with progressive changes in the tissue composition of RV with cardiomyocyte hypertrophy, stress-induced changes in sarcomeric proteins, inflammatory cell infiltration, and changes in ECM.^3^ However, RVD may also specifically affect the composition of the LV. Our proteomic approach has identified distinct sets of 12 proteins differentially expressed in the RV and 6 proteins differentially expressed in the LV of patients with HFrEF with severe RVD. Among 13 upregulated proteins, 6 are components of ECM (FMOD, FBLN5, VCAN, BGLN, MFAP4, and THBS4). Three additional upregulated proteins, namely, UCHL1, MYH10, and MRC2 are intracellular but contribute to ECM remodeling.^6–8^

Our results, thus, suggest that RVD in patients with HFrEF is associated with significant alterations in ECM composition in both ventricles. Propagation of ECM and the presence of regenerative, interstitial, and perivascular cardiac fibrosis are key pathological features of HF contributing to systolic and diastolic impairment,^12,13^ including RVD,^14,15^ and have been proposed as a potential therapeutic target.^16,17^

In patients with HFrEF with RVD, myocardial FMOD and FBLN5 were among the most markedly upregulated proteins, but their upregulation was ventricle-specific (FMOD being significantly upregulated in RV whereas FBLN5 in LV).

FMOD is a leucine-rich proteoglycan that binds collagen and regulates its cross-linking and collagen fibril organization.^18^ In vitro, both cardiomyocytes and fibroblasts can produce FMOD upon proinflammatory stimuli, suggesting that both cell types may participate in the cardiac ECM remodeling.^19^ FMOD together with ECM proteoglycans BGLN and VCAN (both also upregulated in our study) was previously found to be upregulated in the LV in patients with ischemic HFrEF.^20^ Transcriptomic profiling showed an increased expression of the *FMOD* gene in RVD due to pulmonary hypertension.^21^ FMOD protein upregulation has been recently reported in failing RV in patients with pulmonary arterial hypertension.^14^

Along with collagen fibrils, elastic fibers are another important component of ECM. Formed mainly by proteins elastin and fibrillin, elastic fibers provide tissues, especially in lungs, skin, and blood vessels, with elasticity^22^ and play a role in wound healing, especially in the inflammation phase of the process.^22,23^ FBLN5 associates with tropoelastin and assists its deposition into fibrillin-based microfibrils, forming the elastic fibers.^22^ FBLN5 is essential for elastogenesis and is involved in several pathologies including cutis laxa.^24,25^ Expression of FBLN5 is, similarly to collagen fibrillogenesis, regulated by TGF-β (transforming growth factor beta) signaling.^23,26^ Akin to FMOD, FBLN5 was previously found to be upregulated in LV of patients with ischemic HFrEF.^20^

### FMOD and FBLN5 as Biomarkers of RVD

Our initial proteomic analysis (comparing HFrEF patients with severe RVD versus those with preserved RV function) revealed higher FBLN5 and FMOD levels in LV and RV tissues, respectively.

This finding might be biased, however, by lower LVEF and more rapid progression of HF in the severe RVD subgroup of the proteomic cohort.. Nevertheless, analysis of plasma concentrations of FMOD and FBLN5 performed on a larger and independent cohort of patients demonstrated a significant association between the plasma level of both proteins and the degree of RVD.

To the best of our knowledge, this is the first study demonstrating the association between both FMOD and FBLN5, and RV function. As we have used both noninvasive (RVD grade, tricuspid annular plane systolic excursion, and systolic motion of RV basal segment assessed by tissue Doppler imaging) as well as invasive measures of RV function (pulmonary artery pulsatility index and central venous pressure/pulmonary capillary wedge pressure ratio) and received almost identical results, we consider the association between both FMOD and FBLN5, and RV function particularly robust. While FMOD was specific for RVD only, FBLN5 showed also a significant association with RV afterload (assessed as pulmonary vascular resistance) and age. This suggests that compared with FMOD, FBLN5 probably mirrors slightly different pathophysiological processes; RVD might be also a result of chronically elevated RV afterload. However, there was no association of FMOD or FBLN5 plasma levels with LVEF, cardiac output, body mass index, diabetes status, or kidney function. In addition, plasma levels of both proteins were significantly associated with patient outcomes in the Cox proportional hazard model, suggesting that these proteins mirror pathophysiologically relevant processes with clinical significance.

The prognosis of patients with HFrEF is driven by various factors (degree of HF itself, quality of HF treatment, and comorbidities).^5^ All these factors are included in scoring systems used for prognostic stratification (ie, MAGGIC score).^10^ Although the RV function is strongly associated with prognosis in patients with HFrEF as well, it is missing in the MAGGIC score, which is likely due to the lack of a universal single parameter describing RV function. The addition of FMOD to MAGGIC score significantly increased the AUC compared with MAGGIC alone, and the combination of FMOD or FMOD+FBLN5 together with MAGGIC score outperformed the combination of MAGGIC score together with RVD. This suggests that FMOD and FBLN5 do not merely reflect RVD but integrate other (possibly distinct) prognostically important processes. This concept is further supported by the fact that the prognostic role of FMOD and FBLN5 is additive. The additivity of plasmatic concentrations of FMOD and FBLN5 may be attributed to the distinct molecular roles of each protein in the heart (ie, collagen deposition/cross-linking for FMOD and elastic fiber formation for FBLN5) and to a different intensity of the processes in the respective ventricles. However, it should be pointed out that in addition to the heart, FMOD and FBLN5 are expressed in various tissues that may contribute to the plasma pool of both proteins. Some of those tissues are affected by HFrEF (lungs, kidney, liver, or spleen). Vascular ECM remodeling, profibrotic changes, or other molecular alterations in these tissues may, thus, theoretically increase the FBLN5 and FMOD plasma concentrations and contribute to the prognostic value of these proteins in patients with HFrEF. These properties make FMOD and FBLN5 promising blood biomarkers integrating multiple HF-related processes in addition to RVD.

### Limitations

Despite we have matched patients taken for the proteomic analysis (patients cohort 1) with respect to age, body size, HF cause, and comorbidities, those with severe RVD had also markedly lower LVEF and shorter duration of HF suggesting faster HF progression. Although the regression analysis performed on a larger and independent group of patients with HFrEF suggested a robust association between FMOD/FBLN5 and RV function, the potential association of FMOD/FBLN5 with other pathological processes associated with more rapid HF progression (and, thus, more profound RVD) cannot be completely excluded.

We cannot conclude whether the observed FMOD and FBLN5 upregulation in the myocardium of patients with HFrEF with RVD could be attributed to cardiomyocytes, fibroblasts, or other cell types present in the myocardium. Similarly, we cannot determine whether the myocardium is the only tissue responsible for the increased circulating concentrations of the proposed biomarkers.

Our study cohort is considerably male-predominant and included rather young patients with advanced HF reflecting patients referred to our Institute. Consequently, the generalizability of our results to women and to older patients with less advanced HF should be further tested. Most patients in our study were treated by optimal pharmacotherapy/implantable cardioverter defibrillator/cardiac resynchronization therapy device implantation, but some of them underwent heart transplantation or implantation of mechanical circulatory support, which may bias the outcome analysis. As the patients were enrolled before the SGLT2i (sodium/glucose cotransporter 2 inhibitors) era, none were treated with these agents. Although patients were prospectively enrolled, not all of them were followed in our hospital; therefore, the information about changes in pharmacotherapy throughout the follow-up period was not available from all the patients. Similarly, data about further cardiac decompensation events were not available from all the patients, so it was not possible to analyze this end point. As we have strived for maximal applicability of our results, we have included all patients with HF regardless of cause. However, differences in ECM remodeling may exist depending on the HF cause.

## Article Information

### Sources of Funding

This work was supported by the Ministry of Health, Czech Republic, via grants NV19-02-00130 and NU23-01-00323, the Ministry of Education, Youth, and Sports, Czech Republic, via Charles University (Cooperatio Program, research area BIOLOGY and SVV 260637), and the projects of the National Institute for Research of Metabolic and Cardiovascular Diseases (grant LX22NPO5104) and the National Institute for Cancer Research (grant LX22NPO5102) program EXCELES (Excellent Research and Development in the Health Sector), funded by the European Union-Next Generation EU.

### Disclosures

None.

### Supplemental Material

Supplemental Methods

Supplemental Dataset

Table S1

Figures S1 and S2

References 27–31
